# The Hospital. Nursing Mirror

**Published:** 1898-05-07

**Authors:** 


### "The Hospital" Nursing Mirror.

Being the Nursing Section of "The Hospital."

[Contributions for this Section of "The Hospital" should be addressed to the Editor, The Hospital, 28 & 29, Southampton Street, Strand, London, W.C., and should have the word "Nursing" plainly written in left-hand top corner of the envelope.]

### News from the Nursing World.

### THE ROYAL RED CROSS.

The Queen has been pleased to confer the Royal Cross on Mother Patricia and Mother Jacobs for their services to the sick and wounded.

### ROYAL APPRECIATION.

The Queen Regent of Spain gave the members and visitors to the International Congress of Hygiene and Demography recently assembled in Madrid a cordial greeting. Our countrymen have been especially noticed, and her Majesty has expressed her admiration of British nurses in flattering terms, especially of those now on duty in India.

### ANOTHER PLAGUE VICTIM.

Nurse Higgins, a British nurse employed at Hong Kong, has fallen a victim to the plague.

### WAR FEVER.

The war fever runs very high in America, especially amongst women. They crowd to offer themselves for enrolment under the Red Cross banner, unfurled by Miss Barton at the hospital, No. 233, West One Hundredth Street. All classes are represented, and, in spite of the decision of the Government to send only men to the front, large numbers are under instruction.

### THE BUSBY CONVALESCENT HOME.

The new convalescent home for nurses at Busby, near Clarkston, in Renfrewshire, will shortly be opened. Our readers will remember that we gave an account of Dr. Samuel Johnstone Moore's munificent bequest last year, and will look forward with interest to the opening ceremony.

### SUNDERLAND NURSING INSTITUTE.

The report read at the ninth annual meeting of the Sunderland Nursing Institute recorded considerable progress. Since the district nursing has been taken over by the Sunderland District Nursing Association, the institute has been worked on co-operative principles, and since the home has been made comfortable the whole of the nurses' earnings have been divided amongst them. The present staff numbers 23, and is fully employed—an increase of eight since the beginning of last year. The number of cases during the year was 204; the total receipts, including subscriptions, were £823. There is a loss of £14 upon the year's working.

### MEDICAL MISSIONS.

The rise and growth of medical missions was interestingly illustrated at the annual meeting of the Palestine and Lebanon Nurses' Mission at Exeter Hall on April 22nd. The Rev. H. C. Sturdy, M.A., took the chair, and there were present the secretary of the society, Miss Lloyd; the founder, Miss Wordsworth Smith; and two of the mission nurses, Miss Ward and Miss Elliot. Fifteen years ago the fascination of the Holy Land drew Miss Wordsworth Smith and a friend to settle in the highlands of Lebanon, and, devoting themselves to helping the poor, they found themselves forced to provide medical advice and assistance. The house they built for this purpose was soon inadequate. Then Mrs. Meredith, the president of the Princess Mary Village Homes, Addlestone, came to the rescue, and sent trained nurses to their help. Now there are 5,000 patients annually attending the hospital and dispensary, and more beds are needed in the hospital and more nurses and schools in the villages. It would appear that a nurse's training is becoming an essential part of the equipment of a missionary, whilst the power of teaching any of the arts of civilisation is by no means to be despised. Volunteers for this mission may obtain all information from the Secretary, 143, Clapham Road, S.W.

### NOTTINGHAM AND NOTTS NURSING ASSOCIATION.

There are many satisfactory features in the twenty-second annual report of the Nottingham and Notts Nursing Association. Notwithstanding the comparatively cheap rate at which the nurses are supported, the staff is efficient, and a bonus of £205 was divided last year amongst the thirty-seven private nurses. Mr. R. Evans' remark, on moving the report, recognising the arduous nature of a nurse's work and the desirability of increasing their salaries as more money came in, ought to unloose the purse-strings of the subscribers. Contributions increased during the year by £15; but a larger amount is needed, as a twelfth nurse has been added to the district branch and three more are needed. Mr. Samuel Johnson said, in seconding the report, that they had an association in a town of a quarter of a million population doing a vast amount of nursing at a cost of less than £1,000. The deficit in the year's accounts is £87. The association pays its nurses £30 a year, with uniform, and no nurse is placed on the staff without having had three years' training.

### NATIONAL HOSPITAL FOR THE PARALYSED AND EPILEPTIC (ALBANY MEMORIAL).

The Countess of Halsbury, accompanied by Lady Evelyn Gifford, was present at an entertainment to in-patients on Thursday, April 28th, and in the course of the evening presented prizes to probationer nurses, in connection with the annual examination. Mr. Burford Rawlings, secretary and director, opened the proceedings, and alluded to the fact that, in addition to women, some few men were trained, remarking that medical authorities are practically unanimous in favouring the employment of men in certain classes of cases, though it is true that we look for qualities in a nurse which are more readily associated with the feminine nature. He was followed by Dr. Tooth. The following was the prize list—Bandaging: 1st prize, Pro. N. Delphine Stoer; 2nd prize, Pro. N. Hettie Bovey. Theory and application of electrical treatment: 1st prize, Asst. N. Frances M. Cunningham; 2nd prize, Asst. N. Edith

M. Ferguson. Physiology: 1st prize, Pro. N. Caroline Franklin; 2nd prize, Pro. N. Hettie Bovey; 3rd prize, Pro. N. John D. Carnochan. Practical work in the wards and general conduct: Prize, Pro. N. Delphine Stoer. After the presentation, Dr. Buzzard tendered thanks to Lady Halsbury for her presence, and spoke of the long connexion of the Lord Chancellor with the hospital as a member of the board of management. The musical programme, arranged by Miss M. Allanson Winn, was of a high order, and, in addition to Miss Winn, included the following performers: Miss Janotha, Mr. Orme Darvell, Mr. Percy Woodgate (violin), and Madame Mera (accompanist). At the conclusion, the chaplain, Rev. W. R. Greenhill, moved a vote of thanks to Miss Allanson Winn, and the performers.

### THE CHILDREN'S COUNTRY HOLIDAY FUND.

HOLIDAY-MAKING is no longer regarded as a pleasant though unprofitable pastime. Year by year sees the recreation hour and country sojourn recognised more widely as a necessary preventive to illness. As this is so, the more farseeing wish to bestow on the unfortunate ones who hardly know the meaning of the word holiday the same boon as many now enjoy as their natural right. More especially the cry of the little ones appeals to them, and the Children's Country Holiday Fund is its fitting expression. It aims at giving each of the 30,000 city-bound urchins a fortnight's scamper in the green fields or by the sea shore. Men, women, homes, and money are needed; men and women to choose the homes, select the children, and to exercise wise oversight over all; cottage homes to receive the guests, and money to pay expenses. The chairman (Mr. Samuel A. Barnett) and the vice-chairman (Mr. John Haines) will gladly receive contributions and offers of volunteer workers at the office, 10, Buckingham Street, Strand.

### TORQUAY NURSING ASSOCIATION.

THE position of the Torquay Nursing Association is only fairly satisfactory. There has not been the hoped-for development in honour of the Diamond Jubilee, and all the income has been absorbed in the payment of current expenses. As there is a debt of £800 on the building, its friends rightly feel that each year should see some diminution of this burden; whilst, but for a special appeal in October last, the number of district nurses must have been curtailed. But there are hopeful signs to be discerned; an awakening interest in the sick poor is spreading. The "Queen's" inspector reports most favourably upon the work of the nurses, and many of the patients have expressed their gratitude most cordially.

### PRIZE-GIVING AT NORWICH.

AT the conclusion of a series of lectures to women and girls on "Home Nursing," given by the Roman Catholic district nurse at Norwich, a public prize-giving was arranged by the secretary, Mr. Bernard Thorn, in conjunction with a musical entertainment. The Very Rev. Canon Duckett took the chair, and nurses in uniform superintended the seating of a representative gathering. The women have taken the keenest interest in the lectures, and the nurse has had the support of the medical men and clergy of all denominations.

### MELBOURNE DISTRICT NURSING SOCIETY.

IT is a matter of regret to all concerned, and especially to the poor of Melbourne and its suburbs, that, owing to financial disability, the "district" or benevolent branch of the Melbourne District Nursing Society has had to be abandoned for the present. Very nearly 1,000 visits were paid in the course of last year, and 714 cases were attended. On the occasion of the annual meeting Mrs. Maine, who for the last seven years has been hon. treasurer, resigned, and Mrs. Kent Hughes was appointed in her stead. It is sincerely to be trusted that the residents of Melbourne will endeavour to restore the benevolent branch of their nurses' work. It is a wealthy city, but the poor there, as elsewhere, are always present, and in need of alms.

### PARISH NURSING IN LOCHWINNOCH.

ON the occasion of the fifth annual meeting a very earnest appeal was made for further help to the friends of Lochwinnoch Parish Nursing Association. This society, in affiliation with the Scottish Branch of Queen Victoria's Jubilee Nurses' Institute, has at present one nurse, costing £109, with an income which last year only reached £83. In spite of this the work has outgrown the strength of one nurse, and the need of a second is urgently felt on all sides. Miss Sawyer, the nurse, attended 214 cases last year, a large number considering the scattered population.

### NURSING IN DENBIGH.

IT is always a pleasure to record good work, and especially to note its due recognition in the proper quarter. At the annual meeting of the Denbigh Branch of the Queen Victoria's Jubilee Institute for Nurses, very cordial reference was made to Nurse Williams and the excellence of her work, and the announcement by Mr. Gold Edwards, the chairman, that it was proposed to increase her salary by £5, "as a slight acknowledgment of all her valuable services and the hearty good-will she displays in her work," was received with much enthusiasm.

### SHORT ITEMS.

THE poor of Haggerston evidently appreciate the ministrations of the district nurses, since a donation of £1 0s. 4d. has lately been given to the funds, which sum represents the "Lent savings" of a Lads' Guild of Perseverance at Haggerston.—In connection with the Nurses' Home, Newcastle, the committee has established a sick fund towards providing rest and change for those nurses whose health suffers from the unhygienic conditions under which they so often perform their duties. During the past year the committee have divided the sum of £325, bonus-money, among the nursing staff.—Miss Ellis, of the Cumberland Nursing Association, gave an address in the Lecture Room, Cleator, on the subject of district nursing. A committee was appointed at the close, and the secretary empowered to make immediate application for a nurse to serve the district.—One thousand five hundred pounds were voted at a recent meeting of the Committee for Relieving Distress in Crete and Thessaly. Two doctors and two midwives are employed and medicines are distributed. Grants were also made for educational purposes, and relief in kind.—Mr. C. H. Bailey, of the Tyne Engine Works, Barry Docks, has given £20 17s., and his workmen £39 3s., a total of £60, to the Barry Nursing Association.

### Lectures to Surgical Nurses.

By H. A. LATIMER, M.D. (Dunelm), M.R.C.S. of Eng., L.S.A. of London, Consulting Surgeon, Swansea Hospital; President of the Swansea Medical Society; Lecturer and Examiner of the St. John Ambulance Association, &c.

### XXXI. — THE FINAL PREPARATIONS FOR AN OPERATION—TIME AND KIND OF FOOD TO BE GIVEN BEFORE THE ANÆSTHETIC IS TAKEN—CONDUCT OF THE NURSE IN THE OPERATING-ROOM—NURSES' DUTIES AFTER REMOVAL OF THE PATIENT TO BED AND HER TREATMENT OF THE POST-ANÆSTHETIC STATE.

If an anæsthetic is to be given during the operation you must see that the patient has a light meal four hours before the time fixed upon for its performance, and that nothing whatever is taken afterwards. A cupful of some animal broth is the best preparation for him in this respect. Some surgeons do not give any food for many hours before the anæsthetic is taken, preferring to operate early in the morning, and not to allow anything to be taken after the evening meal of the preceding day; but I am sure from personal observation that this is not a wise plan. I have often given anæsthetics for such cases, and do not consider that they are well-borne. People prepared in this way are mostly weak and flatulent. These precautions about food are taken because vomiting is pretty sure to occur if the stomach is occupied when an anæsthetic is taken. Under such conditions sickness is very apt to take place during the period of anæsthesia itself, interfering greatly with the comfort of the person who is giving the ether or chloroform, and with the safety of the patient who is being sent to sleep by one of those agents. Before leaving the bed, see that the patient empties the bladder; and if he should have any false teeth, see that these are removed. If it should be a woman, comb her hair, and secure it by braiding it. Now let a warm dressing gown be donned, and all is prepared for the operating table.

When you have arrived in the operating room, you will be detailed off to perform certain duties by the sister in charge of your ward, or by the surgeon, should the operation be taking place in a private house. These duties are connected with the medical man who is giving an anæsthetic to the patient, or with the surgeon who is operating. In all you do take care to avoid being fussy; move about the room quietly, and avoid being in the way of the medical men who are present. The less noise you make the better for everybody, and especially for the patient. I do not know whether any of you have at any time been put under the influence of an anæsthetic; but I may tell you, as one who has had that experience, that before its influence has been fully exercised on the subject who is inhaling it, noises of all sorts are very distressing and disturbing to him. In the early stage of anæsthesia, before consciousness is lost, vibrations and sounds in a room become accentuated to a painful degree, and a person who is in the semi-conscious state is harmed if quietude is not maintained around him, and is kept longer awake than is at all necessary; and he is also much more liable to struggle and to have interferences with breathing than is the case if his special senses are not irritated in any way. Therefore abstain from loudly drawing water from a tap, or from making clattering noises with cans and basins, and from moving about the operating-room. When the patient is fully under the influence of the anæsthetic which is being administered to him, his special senses of sight, sound, and touch are abolished, and no harm to him will result from inattention to these matters I have been speaking of.

The anæsthetist will require a supply of clean towels and of pannikins to be within his easy reach for use if the patient should be sick, and it will be your duty to see that these are arranged close at hand for him. The surgeons will require a constant supply of sponges for arresting bleeding and mopping up discharges, and you will have to squeeze these out of suitable antiseptic lotions from time to time to be ready for their use; see that you squeeze them almost dry before handing them on, for they must be in that state if they are to prove of any service to them. Do not throw away any tissues which have been removed from the body and which have been placed in small basins by the surgeons, as these are often examined later on to make sure of their character with a view to clearing up some obscure point about the nature of the disease from which an invalid is suffering. Put such things aside in some place where they will not be pounced upon and thrown away by some irresponsible bystander who is apt to commit blunders of this sort and then think all is condoned by his saying that he did not think that he was doing anything wrong, or that there was any value to be attached to "those bits of tumour."

The operation being finished, the patient will be removed to his bed, which is to have been prepared for his reception by the bed-clothes having been turned aside. He is to be laid on his back, with his head and shoulders slightly raised (unless he is in a condition of shock, when the head is to lie level with the bed to allow of blood freely reaching the brain), and a cradle is to be placed over the part which is covered with dressings to ward off the pressure of the superincumbent bed-clothes. You will now have to sit by his bed-side until he has regained consciousness, for the double purpose of helping him if he should be sick, and of preventing him injuring himself or removing his dressings while in the half-conscious state. If he should be sick, assist him by turning his head sideways and placing a small vessel under the mouth; if restless, you will best control him by speaking to him and telling him where he is; for in this stage of his illness he will hardly know what is the matter with him, and will require to be recalled from his dream-like condition to a state of every-day life. It is rare for the sickness to persist for long—two or three hours at the most is the outside of this period of suffering. Different anæsthetics affect the same persons in different ways, and it is probable that there is a suitable one for everybody, could the especial variety that agrees be found. Thus, I have known a man be violently maniacal when put under the influence of nitrous oxide gas, and shortly afterwards go quietly to sleep, without any excitement, when chloroform has been administered. The vapours of chloroform and ether produce an unconscious state in the end, but this condition does not immediately result when they are breathed, as is the case when they are pretended to be used in stage plays, where, immediately a handkerchief is applied to the victim's nose by the stage-villain, his victim succumbs to the influence of the drug, with hardly a struggle; in real life some minutes elapse before loss of sensation is complete, and before this stage is reached it is customary for the inhaler to become excited, and, oftentimes violent. The fact is, he is being made drunk through the lungs instead of the stomach, and the practical point which concerns you is that on recovery from the unconscious condition in which he has been placed he feels the inconvenience and discomforts which attend the emergence from the drunken state, being dizzy, and suffering from headache, nausea, and sickness. If he is kept quietly lying down during his period of recovery he may escape from the act of vomiting; if he raises himself from bed, or moves about and is excited, sickness is pretty sure to occur. Whilst attending him you will also satisfy yourself from time to time that the dressings which have been applied to his wound are retained in their proper position, and that there is no recurring hæmorrhage taking place. When he is quite himself again he may be left for awhile, after having been admonished about being careful to keep quiet, and you can attend to your other duties, merely looking to him from time to time. Henceforth he will probably only require from you the ordinary attendances of a nurse.

### Post-Graduate Clinics for Nurses.

By a Trained Nurse.

### THE NURSING OF CHOREA.—I.

It is very important that nurses who make a speciality of children's cases should be thoroughly conversant with the nursing of chorea. Some years since, a nurse in charge of such a case was merely expected, in a general way, to keep her patient clean, nourished, and amused.

The complexities of medicine and nursing nowadays demand, however, that in undertaking the care of a chorea case she shall bring into this care a great deal of skill and intelligence. The nursing of chorea has become an art, and such cases present many interesting points, both to be learned and practised.

Nervous diseases are at all times most interesting from the nurse's point of view, since these involve a considerable amount of mental as well as physical attention. Choreic cases specially call for moral influence on the part of the nurse, and such influence is the more easy and pleasant since chorea is most usually met with in children between the ages of five and fifteen years. Occurring more frequently in girls than in boys, the nurse will often be brought into contact with a choreic patient who proves most excitable, restless, and exhibiting occasional outbursts of emotionalism or bad temper.

Chorea may sometimes—though rarely—be met with in the adult, in which case it is usually associated with pregnancy, the first symptoms coming on in about the third month. These patients are usually most trying and difficult, but since adult chorea is uncommonly met with, it may perhaps be better to deal with the nursing of this disease from the child-patient point of view.

The first step in the care of acute chorea is to put the patient under soothing and healthful conditions. Plenty of fresh air is most essential, but the nurse must be careful to bear in mind that light acts in many cases as a strong stimulus to choreic and jerky movements. Therefore in the acute stages, when the patient proves specially susceptible to sunlight or strong light generally, he or she will do better in a somewhat darkened room. In no case should the bed of such a patient—or, indeed, that of any nervous subject—be allowed to face the window. Such a position is quite enough in many such cases to materially add to general jerkiness and excitement of the nervous muscular system. The slamming of doors, sudden noises, and abrupt entry of visitors to the room must be equally guarded against. The room of a choreic patient should be as quiet and subdued as though it were the room of a dangerously sick person, with this exception, that the child choreic needs quiet occupation and amusement so as to prevent the mind from preying on itself and the patient becoming too self-conscious.

A calm—perhaps a somewhat stolid—nurse manages chorea best. Her movements will be slower and more rhythmical than those of a more energetic person, and thus she will quite unconsciously exercise a soothing influence over the restless, irritable child with the "perpetual motion" of chorea, the tendency to grimace, tongue protrusion and non-rhythmical inharmonious movements of most members of the body.

It must be remembered that it is most necessary for a patient suffering from acute chorea to be kept absolutely recumbent and at rest, in so far as this can be obtained. Where movement is very violent a feather bed on the floor —if the child is too big for a cot—is the safest arrangement, this being perhaps the only instance where a modern nurse will willingly admit a feather-bed into the sick-room whereof she has charge. In the case of a small child, a cot entirely lined and padded with soft pillows is an obviously comfortable arrangement. Even when the device of the feather-bed on the floor is resorted to, unless some sort of barrier be erected, the child during violent movements will often injure itself badly by jerking from the feathers to the floor. A fortification of bolsters is sometimes fixed all round to prevent this. A device which I have found most useful may be helpful to other nurses. Putting the child on a feather-bed on an ordinary bedstead, I have taken some stout garden netting and drawn this tightly and firmly along the sides of the bed, and across the head and foot, at a distance which prevents the patient's body from coming into contact with the head or foot rail.

Broom handles are as useful in constructing the chorea bed, as in forming a tracheotomy tent. Enclosed entirely with this netting, lined with soft pillows, the child cannot easily hurt itself. But, bearing in mind the old truth that "constant dropping wears away a stone," it is equally true that a child constantly projecting itself even against the softest substances, may become very sore. In addition to the protection afforded by pillows and feather bed, I have sometimes had to clothe a small patient in a pyjama suit made of square sanitary towels sewn together, or to bandage elbows and knees with such pads to prevent the injury to these prominent parts caused by beating them against one another and against other objects. The very thinness of choreic children aids in the formation of sores and bruises, and against the production, in any form, of these, the nurse must carefully guard.

A water-bed is admirable in bad cases, for sores are apt to defy watchfulness, and to appear in violent forms of chorea. The reduced vitality of these patients is another factor favourable to the production of sores and bruises.

Restraining braces and a folded sheet are sometimes used to minimise movements, but experience has made me much more favourable to the other means which I have mentioned to prevent injury.

The skin in chorea is apt to become very dry and imperfect in its action. To counteract this the nurse should occasionally at night anoint the patient with oil over the entire body, and give a warm bath the following morning. Tepid douches to the spine will frequently be ordered, and the nurse should administer these without fuss or excitement. Warm baths prove very restful and soothing to the choreic; cold water, on the contrary, is apt to prove a stimulus to movement, even when used for the hands and face, so that tepid water is preferable. Hot water baths are sometimes used, and these, as well as immersion in warm water, certainly diminish for a time the violence of the movements.

The nurse should take her observations as to the patient's condition in so quiet a manner that the patient will not feel as if under observation. So much self-consciousness usually accompanies chorea that the least suspicion of being watched is calculated to considerably increase the jerky movements and general nervousness.

### Death in Our Ranks.

We regret to note the death of Miss Terry, the district nurse at Yarm, from influenza, after a few days' illness. Miss Terry was trained on the Ockley system, and held a first-class certificate as obstetric nurse from the City of London Lying-in Hospital. Before going to Yarm she had worked as district nurse at Sanghall and Shotwick, near Chester. She was much beloved by all who knew her.

### Meetings on Behalf of the Zenana Bible and Medical Mission.

LORD KINNAIRD presided over two meetings, held in the new hall of the Young Women's Christian Association, at 26, George Street, Hanover Square, on behalf of the Zenana Bible and Medical Mission on the 29th ult. At three p.m. a review of the financial position of the society was given, the speakers being the Rev. A. H. Bowman, the Rev. A. R. Cavalier, Mr. W. T. Paton, and Bishop Thorburn, of the American Episcopal Mission. The usual special appeal for the mission was not issued last year on account of the famine, to relieve which the mission collected £9,000, consequently £5,000 was urgently needed to square accounts and meet all expenses. Subscriptions for this sum, on its need becoming known, flowed in merrily, and at the close of the afternoon meeting promises had been received for all but the last £1,200.

In the evening, at the same hall, a number of excellent lime-light lantern slides brought vivid pictures of the life inIndia, and of the sufferings caused by the plague and famine before a crowded audience. Miss Morley and Miss Poynter, who have been working for upwards of six years at Patna, gave short addresses. All showed the undoubted need for the work, and the welcome accorded the workers. It is only fair to state that every speaker emphatically denied the charge so often brought against missionaries to women in India, that they creep into the Zenanas on false pretexts and then inculcate a knowledge of Christianity. No one enters a Zenana except by invitation, and the purport of the visits is clearly impressed beforehand on the owners.

The work is growing. The committee asks for the income to be increased to £20,000 a year. The expenditure is very low considering the amount and extent of the work, and there is a vast field open for humane enterprise in this most interesting land. The committee, too, declared that if they are assured of financial support, the mission will undertake the care and education of the numerous orphans left by the recent troubles in India. Such an offer ought not to be overlooked, and we trust that there are generous hearts and hands that will make it possible for these desolate little ones to be brought up under the supervision of kindly and judicious English women.

### Our American Letter.

THERE is little of interest just now in the American nursing world. Everyone is preparing for the "Trained Nurses' Educational Exhibit," which was opened on the 25th ultimo, at New York. The general idea is to represent the rise and progress of nursing by means of a series of booths. As no charge has been made for space in the Exposition, or for freightage to a reasonable amount, these expenses having been borne by the Management of the International Health Exposition, this section ought to be well represented. Several important training schools have arranged to be represented.

The nurses graduating in 1897 at the Woman's Hospital, Philadelphia, have decided to endow a bed at the hospital for graduate nurses. The hospital authorities have offered the use of a bed on the following terms:—For the sum of £200 (1,000 dollars), the use of a bed for three months in the year, for £400 (2,000 dollars) six months, or in perpetuity for £500. Another £100 will be needed to furnish and equip the room. Efforts are being made to raise the money, the promoter representing that it is a privilege to give, and that the bed will not be a charity, for the doors of the hospital are already open to its own graduates, but a boon to which the nurses will have a right.

### State Children's Aid Association.

No one who realises the importance of early training can look with unsympathising eyes on the year-old State Children's Aid Society. The knowledge that the most extravagant of all waste is that which dooms little children to life-long ignorance and disease is being driven wedge-like home into the dulness and indifference of a selfish public. The most enlightened and noblest sacrifice most for their children, and is it not but one step further when the rulers of the land insist upon justice being done to the children of the State? It is with great content, therefore, that we read the first report of the State Children's Aid Association, and note the many weighty names upon the committee, recognising amongst them those belonging to men and women who are giving time, energy, and money to promote the progress of a subject in which they are experts. The aim of the association is to obtain individual treatment for children under the guardianship of the State. In order to obtain this, it is working for the abolition of the barrack and workhouse schools, and to have the children educated in family circles; and is also endeavouring to obtain further powers for dealing with neglected children. The first chairman of the society was Lord Peel, and he has been succeeded in this office by Lord Herschell. These gentlemen and the committee over which they preside closely watch legislation on behalf of children. Meetings, too, are held and addresses given, with a view to rousing the public conscience upon the subject. It is owing to the action of the association that the Kirkdale and the Sutton Barrack Schools have been abandoned.

The society mourns the loss of two powerful friends in Mr. Ernest Hart and Sir William Windeyer. They were men whose names inspired confidence, and will recommend the work of the society to many.

### Minor Appointments.

HOMŒOPATHIC HOSPITAL AND PRIVATE NURSING HOME, LANSDOWN GROVE, BATH.—On April 30th Miss E. Thompson was appointed Assistant Matron of this hospital. She was trained at the Royal Infirmary, Preston, and has subsequently been charge nurse in the Royal Infirmary, Bristol; night superintendent at the Paddington Infirmary, London; and night sister at the Cardiff Infirmary. Miss Thompson has recently undertaken holiday duty in several cottage hospitals.

MANCHESTER ROYAL EYE HOSPITAL.—Miss Margaret Seller was appointed Sister of this hospital in February. She was trained at Oldham Infirmary, and has since been district nurse at Woodney, night superintendent West Ham Hospital, and sister of the Seamen's Hospital, Greenwich.—Miss Clara Blackheth was appointed Sister in April. She had just completed her training at Bolton Infirmary.

HAWKHEAD ASYLUM, PAISLEY.—On April 19th Mrs. Christina Freer was appointed Head Nurse of this institution. She was trained at Guy's Hospital, and has previously held the following positions: Matron, the Hosmer Keogh Hospital, Salt Lake City, U.S.A., and under matron, Cane Hill Asylum, near London.

THE PARK HOSPITAL, HITHER GREEN, S.E.—Miss Rose Lindsay, who was at the London Hospital for five years as probationer and staff nurse, has been elected Charge Nurse of this hospital. She has been sister at the Metropolitan Hospital, Kingsland Road, to January of the present year.

ROYAL ALBERT EDWARD INFIRMARY, WIGAN.—Miss Sarah Hopwood, the new Sister of the male wards of this infirmary, received her training at the Blackburn Infirmary. She has been for five years theatre sister at the Royal Hospital, Sheffield.

ACTON COTTAGE HOSPITAL.—Miss Gertrude Willmott Dixon, staff nurse at the Woking Cottage Hospital, has been appointed Staff Nurse here. She holds a three-year certificate from the Royal Hospital, Portsmouth.

SCULCOATES UNION WORKHOUSE INFIRMARY.—Miss Sarah Ward was appointed Superintendent Nurse of this infirmary on April 26th. She was trained at, and was afterwards night superintendent, at the Parish Infirmary, Liverpool.

### Benefits of State Registration for Nurses—Actual and Possible.

"I. N. A.," a registered nurse of Cape Colony, gives vent to the grievances of her class in the March number of the *South African Medical Journal*. Her letter is interesting at this time, when the State registration of nurses is being so strongly put forth as a panacea for all the ills of nursedom, and will afford our more far-seeing nurses food for meditation. The article runs as follows :—

"When the Government registration of trained nurses was introduced into Cape Colony, those of us who were striving to raise the standard of nursing as a profession hailed the step as one likely to help us in our efforts—a measure that would result, we trusted, in good and justice to nurses. Good, because it would define, and therefore strengthen, their professional position; justice, because the women who were really trained were to be, by registration, differentiated from those who merely donned a nurse's dress and were 'helps" in a sick-room. We felt it would a be a benefit, by increasing *esprit de corps*; by binding nurses together in a common interest; a means of bringing home to them that they are not units who can do as they like, but they are bound, not only by the general social obligations which bind us all, but by a grave responsibility to their fellow-workers, who gain or lose as a body in proportion as the action of the individual is good or bad.

"How far have our hopes been realised? The skilled worker, whose efficiency has been recognised and registered by an official body, naturally looks to receive some benefits from the position she has spent time, labour, and money in attaining. What are they? Shorter hours of work? No. The registered nurse in hospital has no shorter hours than the unregistered, and in private nursing a day of at least seventeen hours is expected from her; of work she probably gets through more than her untrained sister, for she knows better how to do it; as to money, the young woman who, having been six weeks a patient in a hospital, and on the strength of such residence passes herself off as a nurse, demands her two or three guineas per week as boldly as the woman who has had years of training. Is it to be wondered at if nurses begin to ask, Why should we trouble with examinations? Why should we give up three years to training, when women who have had none or six months get work as easily and fees as good as we do? It is true that there are penalties attached to one's calling oneself a trained nurse if unregistered, but these are easily avoided. Employers grumble at the "nurse," but do not find out until too late (if ever) that they have been duped into paying for skilled labour when they have had unskilled; and that brings me to the first practical point. There would be some hope of justice to trained nurses if employers would ascertain that the nurse really was what she represented herself to be, and if they refused to pay the same fee to the untrained but possibly excellent person who comes to aid them as the real nurse who has spent years in gaining skill and experience. We look to medical men to help us to obtain this justice. Those who prefer untrained women can still have them, but let them call them untrained, and let them be paid accordingly.

"Nurses' dress is worn by all alike, and even the uniform of an institution is copied by any one who fancies it; but if the Cape Colonial Medical Council would issue some kind of badge, to be worn by the nurses on their registers, the public would have an easy way of distinguishing the registered nurse, and this would be a simple way of stating her professional qualifications."

### Presentations.

Nurse Peterkin, who for the last five years has worked zealously as district nurse at Bridgwater, received a purse containing £25 at the monthly meeting of the Executive Committee. Mrs. Ramsay presented this handsome token of appreciation on behalf of the subscribers, amongst whom were numbered many of those who had been under Nurse Peterkin's care.

### Everybody's Opinion.

[Correspondence on all subjects is invited, but we cannot in any way be responsible for the opinions expressed by our correspondents. No communication can be entertained if the name and address of the correspondent is not given, as a guarantee of good faith but not necessarily for publication, or unless one side of the paper only is written on.]

### QUEEN'S NURSES.

"Another Queen's Nurse" writes that she quite agrees with "Nurse M. J. L.," and thinks it is very hard lines that they should have to give up their badge and medal at the end of their engagement with the Q.V.J.N.I. District nurses have far more risks and difficulties to encounter than those in hospital. Even if they are not allowed to keep their badge, they might at least have the medal. No nurse would think of wearing the badge when she belongs to any other institution.

### A SUGGESTION AS TO PENSIONS.

"Four Queen's Nurses" write: After reading the letter from "Nurse M. J. L." in The Hospital for April 23rd, might we make a suggestion? Would it not be a great inducement to nurses to remain permanently in the Q.V.J.N.I. if a graduated pension were given after 10, 15, and 20 years of service? Very few nurses remain beyond their time of agreement in the Q.V.J.N.I., possibly as there is very small chance of promotion, and very little opportunity for providing for old age as the salaries are small and not progressive.

### STRAW BONNETS.

A "Queen's Nurse" writes: Perhaps some of the readers of The Hospital would be glad to know that they can make their old straw uniform bonnets like new by using "Maypole Straw Hat Polish," although the dark blue is really a more bronze purple blue than the new bonnets. Still for an every-day bonnet (I suppose every nurse keeps a Sunday best) there is certainly a marked improvement on a shabby one. It is but 3d. per bottle. I have done my "rainy day one" and "my second best." My very latest one I had a few months ago has already only seen daylight about four times. It is my very best. I have enough "Maypole" Polish to do other bonnets for my threepenny bottle. I am not an agent for "Maypole," as might at first sight appear.

### NUTRIENT ENEMAS.

"Matron" writes: In answer to query No. 39, I think a nutrient enema is best given by means of 4 oz. glass syringe with about half a yard of rubber tubing attached to glass end of syringe, the other being fitted with bone-nozzle from Higginson's enema syringe. The piston part of glass syringe should be placed in water when not in use.

"Nurse Mac" writes: In answer to query of "Veritas," I have found the following a good way of giving nutrient enemata: Cut a gum elastic catheter (about No. 12) in half. Attach a piece of rubber tubing to catheter, the other end of tubing to a glass funnel. After oiling, warming, and softening catheter in hot water, pour enema into funnel, allowing a little to pass right through to expel all air. Then quickly pinch tube at lowest end, and gently introduce into rectum. The flow may be regulated by still pinching tube between thumb and finger. Before quite all the fluid has passed from funnel, again pinch tubing at lower end to prevent escape of air into rectum. As in some cases a little force is really necessary, I have found a glass syringe instead of funnel very useful. By withdrawing the piston it can be used as a funnel, and then if the fluid does not flow properly (as sometimes it will not), the piston can again be easily introduced without any discomfort of delay to patient.

In answer to "Query for Nurses," asked by "Veritas" in last week's Hospital, "B. A." writes: I always find the best contrivance for giving nutrient enemas is a catheter, attached by a piece of rubber tubing to a glass syringe. Before inserting catheter draw out piston of syringe. When the catheter has been inserted pour the liquid very gently into glass to allow it to run slowly into bowel. If there is more nourishment than will fill the syringe once, continue pouring slowly before it quite empties to prevent air getting in. If by any chance the catheter gets blocked, and the liquid will not run through, it can be very slowly forced through with the piston of syringe. Before drawing out piston to refill the glass, the tubing must be gripped with a pair of "Spencer Wells" forceps to prevent any of the liquid being drawn back. The forceps can also be put on to regulate the flow. A glass funnel will answer the same purpose as the syringe, but without the advantage of the piston to force if necessary.

### QUALITIES THAT SUCCEED.

"A NURSE" writes: In my experience of hospital life there is one point which has often forced itself on me, and one which I think ought to be remedied if nursing is to be improved. Some hold that nursing and nurses have improved at a great pace within the last fifteen or twenty years. I do not say this is not so. In many ways improvements have been made; but there has also been a degeneration, although none but nurses may have seen it, and it has even failed to make itself apparent to some of them. There are a great many noble women in the profession now, who enter it from the highest motives, but there are also a few who are, at least, vulgar and ignorant. Why is it that these coarser women frequently get on and are soon placed above the refined and sensitive women, who are willing to do good work in a quiet way, but whose lives are made miserable by this undercurrent of vulgarity and taunting, while they cannot lower themselves to quarrel like their charge nurses. No, they rather endure this miserable life until sometimes their health gives way, or they can do it no longer and are forced to give it up. Thus some of the best nurses are lost to the profession, and the fault is always laid on those who leave, not with those who have caused their leaving, either through ignorance or jealousy. So long as this state of things continues, nursing will degenerate, because the worst will be chosen as staff nurses, sisters, or matrons. The refined and sensitive will be driven out of the field, and only the ones who can drive and scold and wield their power in a loud, commanding way will get on.

⁂ "A Nurse" lays a very grave charge against those of her own profession who succeed. Are not jealousies quite as prevalent amongst subordinates as amongst those who succeed? Who can be more selfish than a woman who is a slave to her sensitive nature? If self-forgetfulness be an important quality in a nurse, is it compatible with feelings that are for ever being wounded? It is not everyone who can be a general, and if the special qualities that make a general or a matron are sometimes allied with a coarse nature, it is the qualities, not the nature, that win success, and the possessor of a sensitive heart is not for that reason necessarily fit to be a ruler.—ED. *T. H.*

### NIGHT NURSING IN WORKHOUSE INFIRMARIES.

"L. D." writes: I have read with much interest the paragraph in your last issue regarding the bad management at Coventry Infirmary, and, having had a similar experience myself, shall be glad of space to make known my grievance. I have recently left a small workhouse infirmary, where for two years I did alternate day and night duty of three months' intervals, and during that time I think I can safely say I never enjoyed one single day of quiet uninterrupted sleep. My bedroom was on the ground floor and in a direct line of traffic, for one had to pass it to get to the nurses' common sitting-room, which was next door. In consequence of this there were people passing and repassing all day long, and, be they ever so quiet, footsteps will sound on a bare-boarded floor. Sometimes there were visitors, who had a way of talking all the way along the passage, and sometimes standing outside the door; but after all this was a minor evil compared to the noise overhead, as there was a small ward nearly always in use, the staircase leading to it being only separated from my room by a thin partition. One day, in despair, I tried to get some sleep in the room allotted to me for day duty, but that failed, for underneath was the nursery and overhead the children's dormitory. Then I was audacious enough to speak to the Guardians, and was calmly informed in language more forcible than polite, that "if I did not like it I could go." These worthy gentlemen seemed to look upon all nurses as a sort of machine, "wound up and warranted to go," and to say one felt tired was evidently too great a presumption. The nursing, too, was not of the lightest, there being about 70 patients, many of these being quite helpless. To stand such a strain for long together is beyond anyone's power of endurance. But, now, *nous avons changé tout cela.* I am on night duty in my present post, but, thanks to the kindness and foresight of the matron, I am able to enjoy peaceful sleep, just as I should at night. There is no traffic past my door, people are forbidden into the room overhead in my sleeping hours, and by the aid of curtains and cork carpet all sound is deadened outside. I wake up refreshed and ready for duty, and of course cannot help comparing my present quarters with my last. There must be night nurses, and it is only a matter of common justice to treat them properly and provide suitable quarters for them. Surely it can be done, not without trouble perhaps, but a very little outlay of money, which at any rate would be well spent, and earn the grateful thanks, I am sure, of many of my hardworking sisters in the profession, whose treatment makes one blush for the so-called enlightenment of the present day.

### New Cottage Hospital and Nurses' Home at Acton.

A NEW cottage hospital has just been built at Acton, one more useful monument to the generosity of Mr. Passmore Edwards, by whom most of the expense of erection has been defrayed. The formal opening, at which the Bishop of London and Mrs. Creighton were present, took place on Wednesday, May 4th. The hospital provides room for 10 beds in two main wards, one for men and one for women, of four beds each, and two small single bedded wards for special cases. The women's wards are at one end of the building and the men's at the other, coloured glass doors shutting them off from the central portion. On either side of the front entrance are the matron's sitting-room, and a room which at present will be used as a committee-room; behind these are the kitchen and offices, a dining-room for the nurse, and a theatre. On the two upper floors are the bedrooms for the nursing staff, nice little rooms, simply but comfortably furnished, a bath-room, store cupboards, box-room, &c. It is a pity that the theatre is not larger. The out-patient department also seems somewhat cramped. The hospital will not be in working order for a week or two. Miss Agnes M. Jackson has been appointed matron, and she will have a staff nurse and a probationer under her, while two district nurses are eventually to make their home in the building. In South Acton there is a large poor population amongst whom the new hospital is sure to do a valuable work. The site is a good one, just off the Uxbridge Road, on high and open ground, and contains a good-sized garden. Mr. Carrington Smith, who has taken much interest in the hospital, is the chairman of the committee.

### Appointments.

### MATRONS.

NORTH LONDON HOSPITAL FOR CONSUMPTION.—Miss Jessie Whitton has been appointed Matron of this hospital. She was trained at "Her Majesty's" Hospital, Stepney (in connection with Dr. Barnardo's Homes), where she remained as sister for nearly two years. For the last five years she has been sister-in-charge of Friedenheim, the Home for the Dying.

THE HOSPITAL FOR INFECTIOUS DISEASES, LEIGHTON BUZZARD.—Miss Mary A. Plough was appointed Matron Nurse on April 23rd. She was trained at the Royal Surrey County Hospital, and has been charge nurse at the South-Eastern Hospital, New Cross.

THE NEW HOSPITAL FOR WOMEN, EUSTON ROAD.—Miss Edwards, who was trained at St. Bartholomew's, was appointed Matron of this institution on the 28th ult. She has lately held the post of assistant matron at the Chelsea Hospital for Women.

ST. MARY'S HOSPITAL, MANCHESTER.—Miss Rose Smily, who was trained at Guy's Hospital, and who has up to the present been on the private staff there, has been appointed Assistant Matron of the above hospital.

### For Reading to the Sick.

"RISE up, my love, my fair one, and come away. For, lo, the winter is past, the rain is over and gone; the flowers appear on the earth; the time of the singing of birds is come, and the voice of the turtle is heard in our land."—*Song of Solomon* ii. 10, 12.

**Verses.**

Now the Queen of seasons, bright
With the Day of Splendour,
With the Royal Feast of feasts,
Comes its joy to render;
Comes to glad Jerusalem,
Who with true affection
Welcomes in unwearied strains
Jesu's Resurrection.

Alleluia now we cry
To our King Immortal.
Who triumphant bursts the bars
Of the tomb's dark portal;
Alleluia, with the Son,
God the Father praising;
Alleluia yet again,
To the Spirit raising.

Am I wrong to be always so happy? This world is full of grief;
Yet there is laughter of sunshine, to see the crisp green in the leaf.
Daylight is ringing with song-birds, and brooklets are crooning by night,
And why should I make a shadow where God makes all so bright?
Earth may be wicked and weary, yet cannot I help being glad;
There is sunshine without and within me, and how should I mope or be sad?
God would not flood me with blessings, meaning me only to pine
Amid all the bounties and beauties He pours upon me and mine;
Therefore will I be grateful, and therefore will I rejoice;
My heart is singing within me! sing on, O heart and voice!
—*Walter Smith.*

Be thou content if on thy weary need
There falls a sense of showers and of the spring:
A hope that makes it possible to fling
Sickness aside, and go and do the deed.
For highest aspiration will not lead
Unto the calm beyond all questioning.—*MacDonald.*

That which thou soweth is not quickened, except it die: and that which thou sowest, thou soweth not that body that shall be, but bare grain, it may chance of wheat, or of some other grain. But God giveth it a body as it hath pleased Him.—1 *Corinthians* xv. 36-38.

What is spring after winter but Nature speaking of the Resurrection of her Lord. It is the season when day is lengthening and mastering the night; light is overcoming darkness, and life springing out of apparent death; as in the returning presence of Him who is very life and very light, and maketh all things new. Thus as morning and spring return again and again, so, after the manner of Christ's Resurrection, and in the Image and Likeness of God, must men arise, and be renewed day by day, while day by day the outer man perishes.—*Isaac Williams.*

Spring is now with us—the season when all Nature bursts into fresh beauty, and every day discloses new signs of the Almighty's goodness to His creatures. Should we not remember then that autumn and winter—the seasons of decay and suff-ring—are but the forerunners of this glorious spring, and that the autumn of our lives and the trials of ill-health are but the natural precursors of the everlasting spring and summer which await us all—if we will?—*A. R. P.*

### Notes and Queries.

**The contents of the Editor's Letter-box have now reached such unwieldy proportions that it has become necessary to establish a hard and fast rule regarding Answers to Correspondents. In future, all questions requiring replies will continue to be answered in this column without any fee. If an answer is required by letter, a fee of half-a-crown must be enclosed with the note containing the enquiry. We are always pleased to help our numerous correspondents to the fullest extent, and we can trust them to sympathise in the overwhelming amount of writing which makes the new rules a necessity. Every communication must be accompanied by the writer's name and address, otherwise it will receive no attention.**

*Money.*

(50) Kindly tell me if there is any fund from which nurses can borrow money at a small rate of interest, to be returned by monthly instalments.—*Nurse E.*

The Junius S. Morgan Benevolent Fund, 28, Finsbury Pavement, E.C., is the only one. Its privileges are confined to members of the Royal National Pension Fund for Nurses. If "Nurse E." is not already a member of the Pension Fund she would be wise to join it at once. In discussing the many advantages offered by this fund nurses are apt to forget the benevolent side of the matter conferred by the powerful and wealthy Junius Morgan Fund.

*Hospitals for Nervous Diseases.*

(51) Are there any private homes or hospitals in London for patients suffering from nervous disorders?—*Nurse Marion.*

The National Hospital for the Paralysed and Epileptic, Queen's Square, Bloomsbury, receives private patients. See our advertisement columns for private institutions.

*A Mill Hand.*

(52) Will the fact of having worked in a cotton mill debar a woman's training as a nurse? She for whom I ask has a strong natural bent that way.—*Minister.*

Nothing debars a suitable woman from becoming a nurse excepting delicate health. There is now a great opening in the Poor Law infirmaries for hospital-trained nurses, and as there is still much to be done to raise the nursing in these institutions to the level of the voluntary hospitals it affords ample scope to those who wish to make nursing a means of divine service. Honnor Morten's 'How to Become a Nurse" (2s. 6d. The Scientific Press, London) will give full information as to training.

*The "Asylum News."*

(53) Could anyone kindly let me know where or how the *Asylum News* may be got?—*Mental Nurse.*

The *Asylum News* is published at the Lancashire County Asylum, Lancaster.

*Monthly Nurses*

(54) Could you kindly tell me if there is any institute one could go to after training in monthly nursing? (2) Also whether six weeks' training is long enough without previous experience?—*Frances.*

The Midwives' Institute, 12, Buckingham Street, Strand. (2) Not in the opinion of those who know most about it.

*Delicate Children.*

(55) I have been ill with rheumatism, and it is unlikely that I shall be able to resume nursing for some time. Do you think that, as I am fond of children and have studied music and German in Germany, that I could obtain the care of delicate children in my own lodgings? I am also a good needlewoman.—*Indian.*

It is, of course, possible; but is it wise to incur the responsibility? If you do so you must be well paid—£150 for one child, or £100 apiece for two. But why not undertake the care of a delicate child in its own home as nursery governess? You might then earn £45 and upwards and be comfortably provided for into the bargain.

*The Supply of Plague Nurses.*

(56) Are any more nursing sisters required for plague duty in Bombay?—*A. C. S. (Matron).*

Apply to the Revenue Secretary, the India Office, Whitehall. If accepted, you will be put on the list of reserve nurses, and may be called upon if more are required. At present the lists of those on active service are full.

*Salary.*

(57) What salary ought I to have for walking an hour with a mentally deficient but gentle and agreeable young lady five times a-week?—*N. D. L.*

It entirely depends upon what the lady's friends offer, and what you are disposed to take. Would not 1s. a walk be fair?

*Superfluous Hair.*

(58) Kindly tell me the address of the best place for having superfluous hair destroyed by electrolysis, and give me an idea of what the fee would be?—*Nurse L.*

Although electrolysis is a surgical operation, it is frequently performed by unqualified persons; it is impossible to give either recommendations or a definite scale of charges. Perhaps some of our readers would help "Nurse L."

*In Touch.*

(59) What can a private nurse do to prevent herself getting out of touch with hospital work—in fact, less rusty?—*Mac.*

Join the Commemoration Club, 28 & 29, Southampton Street, Strand, and attend the post-graduate lectures given there from time to time.

*Convalescent Home.*

(60) *E. H. B.* is asked to read the notice with regard to anonymous correspondents at the head of this column.

*Convalescent Aid.*

The Editor begs to inform 'L. J. G." that he has forwarded 1s. to Nurse Agnes, as requested.

